# The By-Band study: gastric bypass or adjustable gastric band surgery to treat morbid obesity: study protocol for a multi-centre randomised controlled trial with an internal pilot phase

**DOI:** 10.1186/1745-6215-15-53

**Published:** 2014-02-11

**Authors:** Chris A Rogers, Richard Welbourn, James Byrne, Jenny L Donovan, Barnaby C Reeves, Sarah Wordsworth, Robert Andrews, Janice L Thompson, Paul Roderick, David Mahon, Hamish Noble, Jamie Kelly, Graziella Mazza, Katie Pike, Sangeetha Paramasivan, Natalie Blencowe, Mary Perkins, Tanya Porter, Jane M Blazeby

**Affiliations:** 1 Clinical Trials and Evaluation Unit, School of Clinical Sciences, University of Bristol, Bristol, UK; 2 Taunton and Somerset NHS Trust, Taunton, UK; 3 Southampton University Hospitals NHS Trust, Southampton, UK; 4 School of Social and Community Medicine, University of Bristol, Bristol, UK; 5 Economics, Health Economic Research Centre, University of Oxford, Oxford, UK; 6 School of Sport and Exercise Sciences, University of Birmingham, Birmingham, UK; 7 Faculty of Medicine, University of Southampton, Southampton, UK; 8 University Hospitals Bristol NHS Trust, Bristol, UK; 9 The Bariatric Group, Nuffield Health Taunton Hospital, Taunton, UK

**Keywords:** Complex obesity, Gastric band, Gastric bypass, Integrated qualitative research

## Abstract

**Background:**

The prevalence of severe and complex obesity is increasing worldwide and surgery may offer an effective and lasting treatment. Laparoscopic adjustable gastric band and Roux-en-Y gastric bypass surgery are the two main surgical procedures performed.

**Design:**

This open parallel-group randomised controlled trial will compare the effectiveness, cost-effectiveness and acceptability of gastric band (Band) versus gastric bypass (Bypass) in adults with severe and complex obesity. It has an internal pilot phase (in two centres) with integrated qualitative research to establish effective and optimal methods for recruitment. Adults with a body mass index (BMI) of 40 kg/m^2^ or more, or a BMI of 35 kg/m^2^ or more and other co-morbidities will be recruited. At the end of the internal pilot the study will expand into more centres if the pre-set progression criteria of numbers and rates of eligible patients screened and randomised are met and if the expected rates of retention and adherence to treatment allocation are achieved. The trial will test the joint hypotheses that Bypass is non-inferior to Band with respect to more than 50% excess weight loss and that Bypass is superior to Band with respect to health related quality of life (HRQOL, EQ-5D) at three years. Secondary outcomes include other weight loss measures, waist circumference and remission/resolution of co-morbidities; generic and symptom-specific HRQOL; nutritional blood test results; resource use; eating behaviours and adverse events. A core outcome set for reporting the results of obesity surgery will be developed and a systematic review of the evidence for sleeve gastrectomy undertaken to inform the main study design.

**Discussion:**

By-Band is the first pragmatic study to compare the two most commonly performed bariatric surgical procedures for severe and complex obesity. The design will enable and empower surgeons to learn to recruit and participate in a randomised study. Early evidence shows that timely recruitment is possible.

**Trial registration:**

Current Controlled Trials ISRCTN00786323.

## Background

The prevalence of obesity in adults is increasing around the world. In the United Kingdom (UK) rates have trebled during the past 25 years to around 24% [1]. If trends persist, 36% of men and 28% of women aged 21 to 60 will be obese in 2015, and worldwide approximately 700 million adults will be living with the condition [1-3]. The prevalence of complex obesity (clinically defined as a body mass index (BMI) of 35 kg/m^2^ or more with co-morbidity or a BMI over 40 kg/m^2^ without co-morbidity) is also on the increase, and the UK prevalence has been estimated at around 2.1% [4,5]. Obesity is associated with a number of health problems, including type-2 diabetes, cardiovascular disease, musculoskeletal disorders, infertility, and psychiatric disorders. The mortality rate for those with complex obesity is more than double that for the general population [6]. Additionally, obesity is an intermediary to social inequalities in health [7] and places a huge financial burden on the National Health Service (NHS). The direct costs of treating diseases associated with being overweight and obesity were estimated at £3.2 billion in 2002, or nearly 5% of total NHS expenditure [8]. On an individual level, living with obesity has been associated with psychological distress and social stigma, both of which may have a significant impact on individuals’ quality of life [9,10]. The prevention and treatment of obesity is thus a key priority for the NHS, and the provision of weight management services for adults is now firmly established as a core policy objective.

Reversal of obesity is uncommon without intervention [11], and the National Institute for Health and Care Excellence (NICE) guidance states that health authorities should establish comprehensive care pathways for addressing being overweight and obesity within their populations, which should include access to diet and exercise interventions, anti-obesity drugs, and in some circumstances weight reduction surgery [12]. However, it is known that many interventions for obesity fail and bariatric surgery is increasingly being viewed as a solution to weight loss, particularly for those with complex obesity. Although surgery is usually considered after patients have attempted other forms of weight loss without success, the exception to this is for adults with a BMI > 50. NICE guidelines recommend surgery as a first-line option for this group of patients (instead of lifestyle interventions or drug treatment) if surgical intervention is considered appropriate. Surgical procedures for those with obesity aim to bring about a sustained reduction in weight through restriction of intake, malabsorption of food and/or hormonal influence. There are several different operations in use including laparoscopic Roux-en-Y laparoscopic gastric bypass (Bypass), laparoscopic adjustable gastric band (Band), biliopancreatic diversion and its duodenal switch variant, vertical banded gastroplasty and sleeve gastrectomy.

Despite the variety of different surgical procedures available, the two most commonly performed operations worldwide for many years have been gastric Bypass and gastric Band. At the time the By-Band study was conceived and set-up, these two procedures together accounted for approximately 80% of all obesity operations in the UK and the United States [13,14]. A gastric Bypass involves restricting the volume of food which can be eaten by creating a small thumb-sized pouch from the upper stomach and a bypass of the remaining stomach. Bypass alters physiology and anatomy in such a way as to achieve early and generally rapid weight loss but has risks of serious early morbidity and, rarely, death [15,16]. Longer term complications may include the need for re-operation because of the development of internal hernias or intestinal obstruction and nutritional deficiencies. A gastric Band is an inflatable silicone device which is placed around the top portion of the stomach to create a smaller stomach pouch. The Band also achieves weight loss, but this is generally more gradual. Short term surgical risks of a Band are very uncommon [15] but longer term complications include band erosion or migration, pouch dilatation, leakage from the circuit or infection which may require revision surgery or band removal [16,17]. Over the past three years, interest in sleeve gastrectomy has increased significantly and indeed recent UK individual surgeon data shows that rates of sleeve gastrectomy have now surpassed those of gastric Band in the NHS [18]. Data from a worldwide survey show similar patterns of change [19]. Sleeve gastrectomy reduces the stomach to about 25% of its original size, removing a large portion of the stomach along the greater curvature, leaving a sleeve or tube-like structure. Complications include sleeve leakage, blood clots and infections, nausea and vomiting.

Evidence for the different types of surgery has been summarised [11] and updated. There were 20 randomised controlled trials (RCTs) identified in the review that compared different types and variations of bariatric surgery, with only one comparing gastric Band with gastric Bypass. The latter single centre trial in Italy included 51 participants, excluded some after randomisation, did not analyse the data according to the intention-to-treat principle, did not blind outcome assessors and did not assess health-related quality of life (HRQOL) [20,21]. By updating this review, we identified another six trials comparing different bariatric procedures and one further trial of gastric Band and Bypass surgery [22]. This study randomised 250 patients, but differentially excluded some after randomisation creating imbalance in the numbers in each group and an imbalance in key patient characteristics at baseline (age and BMI) and the analysis was not by intention-to-treat [22]. The generation of the allocation sequence was unclear; there was incomplete outcome data at follow-up and no details of the number of participants completing HRQOL questionnaires. The evidence of the relative effectiveness of Band and Bypass is, therefore, inadequate with just two single centre trials that have an uncertain risk of bias and inadequate data characterising HRQOL. The availability and choice of bariatric surgical practice is consequently based on the preferences of local commissioners, surgeons and patients and there remains an urgent need for a well-designed RCT with clinically relevant comparisons, measures of generic and disease specific HRQOL, cost-effectiveness evaluations and at least medium term follow-up and documentation of longer term adverse events. This need was highlighted in the systematic review, but it was also stated that a trial of gastric Band versus Bypass may be too difficult to conduct and recruit into because of strong preferences amongst surgeons that influence patient selection for surgery [11]. The By-Band study (funded in 2011), therefore was designed to compare the two most commonly performed bariatric operations at that time (gastric Band and gastric Bypass). It was designed in two phases so that the barriers to effective recruitment could be overcome within the first phase (an internal pilot), before extending into the main trial (Phase 2). A review of the literature and of current UK practice regarding sleeve gastrectomy will also be undertaken in Phase 1 to understand whether uptake and standardisation of the procedure is sufficient to extend the study in Phase 2 to include a third treatment arm.

## Methods/Design

### Aims and objectives

The By-Band study will compare the effectiveness, cost-effectiveness and acceptability of Band versus Bypass surgery for treatment of complex obesity. We will test the joint hypotheses that (a) Bypass is non-inferior to Band with respect to excess weight loss of more than 50% at three years and that (b) Bypass is superior to Band with respect to HRQOL at three years. In the primary analysis, both outcomes will be considered collectively, that is both hypotheses must be supported to conclude that Bypass is more effective than Band.

Specific objectives are to estimate:

1. The difference between groups in the proportion of patients achieving > 50% excess weight loss at three years;

2. The difference between groups in their average EQ-5D health state score [23] at three years;

3. The difference between groups with respect to a range of secondary outcomes;

4. The cost-effectiveness of gastric Band and gastric Bypass.

### Study design

By-Band is a pragmatic RCT with two phases.

**Phase 1** will establish whether it is possible to recruit into this surgical trial using integrated qualitative methods in two centres. It will develop of a core outcome set to assess the benefits and adverse events of obesity surgery and undertake a literature review and review of current practice of sleeve gastrectomy. Progression to Phase 2 will depend upon the results of these initiatives and will be reviewed with the Trial Steering Committee (TSC) and the funder, National Institute for Health Research Health Technology Assessment Programme (NIHR-HTA).

**Figure 1:**
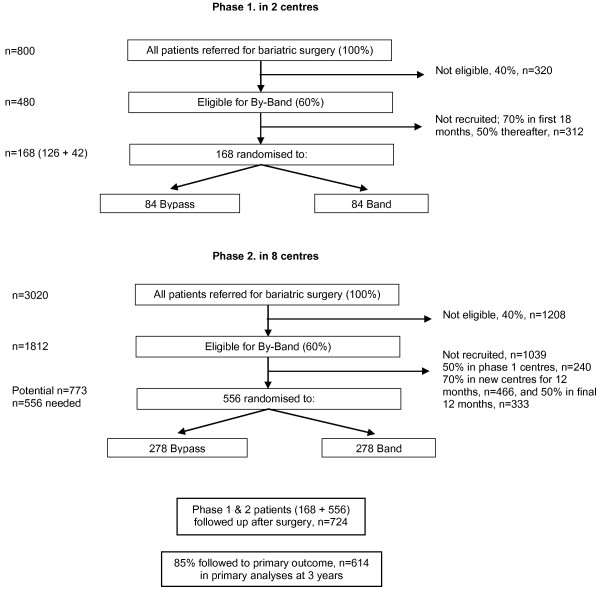
**Trial schema.** Schema showing the number of patients to be recruited in Phase 1 (two centres) and Phase 2 (eight centres), with the anticipated eligibility, recruitment and follow-up rates.

**Phase 2** will extend recruitment to up to six additional centres, using the optimum methods of recruitment established in Phase 1. The overall schema for the trial is shown in Figure 1.

### Research approval

Research ethics approval was granted by the South West - Frenchay Research Ethics Committee (reference 11/SW/0248) in December 2011.

### Study population

All patients referred for bariatric surgery will form the target population and be screened for trial eligibility. Male and female patients > 18 years of age referred for bariatric surgery according to NICE guidelines (BMI of 40 kg/m^2^ or more, or BMI of 35 kg/m^2^ or more and other co-morbidities that could improve with weight loss), who are fit for anaesthesia and surgery, committed to follow-up and who provide written informed consent will be eligible. Ineligible will be participants with, i) a history of previous gastric surgery, ii) a history of surgery for complex obesity, iii) large abdominal ventral hernia, iv) Crohn’s disease, v) liver cirrhosis and portal hypertension, vi) systemic lupus erythematosus, vii) known silicone allergy and viii) participants who are pregnant (women who have given birth and women planning pregnancy will NOT be excluded). We will also record specific reasons where surgeons are unwilling to randomise a patient.

All reasons for ineligibility will be recorded in the trial screening log and where eligible patients decline randomisation, we will ask for written consent to access clinical records to collect baseline details, the type of surgery performed and to invite them for one assessment of weight and HRQOL at three years (the primary endpoint). The screening log will be reviewed on a monthly basis to provide feedback to recruiters since it will help to understand surgeons’ and patients’ preferences for types of surgery. It will also allow the trial results to be reported in accordance with the CONSORT guidelines.

Eligible patients will be informed about the trial and given the patient information leaflet and an appointment for a ‘recruitment consultation’. At that consultation they will be given the opportunity to ask questions about the study and treatments, and invited to participate in the trial. These consultations, where consent is given, will be routinely audio-recorded and form part of the qualitative study.

### Randomisation

After trial eligibility has been confirmed and consent given, randomisation will be carried out. This will be usually within a week of gaining patient consent. A secure Internet-based randomisation system will be used ensuring allocation concealment. The allocation will not be revealed until participant details have entered into the system. Participants will be allocated in a 1:1 ratio and cohort minimisation (with a random element incorporated) will be used to ensure the groups are balanced for diabetes (any type/none), and BMI (more than 50/50 or less). Allocation will also be stratified by centre.

Patients will be informed about their randomisation arm as soon as possible and to allow them time to make arrangements for support at home after discharge from hospital (which is different between the two procedures) and it will allow surgeons time to efficiently plan an operating list (because of the time difference required in theatre for each procedure). We have considered the risk of withdrawals after randomisation and before surgery and this will be monitored closely, but we are anticipating that with feedback from the qualitative research, the recruitment process will have created equipoise among participants.

### Integrated qualitative research

The integrated qualitative study aims to understand the recruitment process and work with study staff to put any necessary changes in place to optimise this. The process of recruitment will be explored using the following methods based on previous work by Donovan *et al*. [24]:

(a) Patient pathway through eligibility and recruitment: a comprehensive process of logging of potential RCT participants through screening and eligibility phases will be used to monitor recruitment. Flow charts will be produced to show the degree of complexity of participation and any variations between centres.

(b) In-depth semi-structured interviews to understand perspectives of participation will be conducted with three groups:

i) Members of the trial management group (TMG), including the chief investigator (CI) and those most closely involved in the design, management, leadership and coordination of the trial;

i) Clinical and recruitment staff at the participating centres;

i) Eligible participants, including those who agree or decline to take part. Interview topic guides based on guides used in previous trials [24-26] will be used to ensure similar areas are covered in each interview within each group, but also encouraging the informants to express their own views about the RCT and any recruitment challenges expected or experienced.

Interviews will be audio-recorded and transcribed and analysed thematically using techniques of constant comparison and case-study approaches [24,25]. Interviews will provide data about: the perspectives of eligible patients; the evidence underlying the RCT, including the importance of the question and the commitment of staff to it, as well as individual clinical equipoise; the application of the protocol in clinical centres and any logistical issues; and suggestions about reasons for recruitment difficulties and potential solutions from those working closely within the RCT.

(c) Audio-recording of recruitment appointments: all staff involved in providing information about the RCT will be requested to audio-record appointments where they provide information to patients and attempt to recruit them to the RCT.

Audio-recordings of appointments will be transcribed *verbatim* whole or in selected parts, as necessary for analysis. Appointments will be analysed thematically and with the techniques of focussed conversation analysis - pioneered in previous studies [24] - to identify and document aspects of informed consent and information provision that are unclear, disrupted or hinder recruitment. Recordings will be listened to by JLD and SP only. An assessment will be made as to whether the appointment is recruiter or participant-led, and also the degree to which there is evidence that the participant has understood the key issues of equipoise, randomisation, participation in the RCT, the option to choose their treatment, and the option to withdraw from the research at any time. These data will form the basis for feedback to individuals and to determine the content of the information, and training programmes to be initiated in Phase 2 of the RCT.

(d) Non-participant observation of staff-patient interactions: observations of clinics such as the ‘one-stop’ and pre-assessment clinics and ‘information days’ will be undertaken with consent. The aim of the observations will be to provide additional information about the recruitment process.

### Implementation of qualitative findings

The qualitative researcher (SP) will undertake the data collection for (a) to (d) in each clinical centre, and lead the analysis of the data with supervision and assistance from the qualitative study lead (JLD). Data will be analysed first after approximately two months of active recruitment and the key anonymised findings from each of (a) to (d) presented to the CI and staff in the clinical centre, with confidential feedback also provided to individual recruiters. Selected relevant findings from (a) to (d) will be used to develop a ‘plan of action’ with the CI and principal investigators (PIs) to improve recruitment and determine further data collection and analysis to evaluate the implementation of the plan. This cycle will be repeated as required. Aspects of the ‘plan’ in other similar trials Recruitment will be up-dated regularly and any changes noted. Issues discussed in other studies included streamlining patient pathways, removing logistical barriers, and improving the presentation of generic and specific study information [25]. Minutes of meetings will document salient recruitment issues, and these and recruitment levels, will be discussed at TMG meetings.

### Progression from Phase 1 to Phase 2

The criteria for progression to the full trial will be reviewed at 18 months after recruitment has started. The aim is to achieve i) 60% of patients referred for bariatric surgery to be eligible for the trial (if necessary revising the eligibility criteria), ii) 50% consent to randomisation, iii) < 5% failure to receive allocated treatment, and, iv) < 5% lost to follow-up (average duration of follow-up will be nine months at this point). In addition, a review of the literature regarding sleeve gastrectomy and a review of current UK practice of sleeve gastrectomy will be undertaken to understand whether uptake and standardisation of the procedure is sufficient to extend the study in Phase 2 to include a third treatment arm.

### Trial interventions

Wherever possible, after randomisation, participants will be listed for surgery within 18 weeks of referral for surgery, unless specifically requested by the participant for personal reasons. The protocol for the surgery is pragmatic. Both surgical procedures will be performed laparoscopically under general anaesthesia with all patients receiving antibiotic and deep vein thrombosis prophylaxis.

### Laparoscopic adjustable gastric banding (Band surgery)

The procedure will involve placement of laparoscopic ports, creation of a pneumoperitoneum and placement of retractors as the surgeon chooses. The choice about the type and size of adjustable gastric band will be made by the surgeon. If a hiatal defect is present it will be repaired and this recorded. Closure of a pre-existing umbilical hernia or concomitant cholecystectomy will be carried out at the discretion of the surgeon and recorded. It is considered mandatory to dissect the lesser curve using the ‘pars flaccida’ technique, to use gastro-gastric tunnelling sutures and to fix the port to the anterior abdominal wall. An apronectomy is prohibited.

### Laparoscopic gastric bypass surgery (Bypass surgery)

The laparoscopic ports, creation of a pneumoperitoneum and placement of retractors may be performed as the surgeon chooses. Creation of the biliary and gastric limbs and formation of the gastric pouch is performed as the surgeon chooses. Upper limits of 75 cm and 150 cm are recommended for the biliary and gastric limbs, respectively. Testing integrity of the anastomoses, closure of a pre-existing umbilical, internal hernia or hiatal hernia defect or concomitant cholecystectomy will be carried out at the discretion of the surgeon and recorded. Formation of a horizontal gastric pouch that includes the fundus and undertaking an apronectomy are prohibited.

After surgery, patients will be nursed within a specialist ward and oral intake commenced according to the local policy and at the surgeon’s discretion. The day of discharge will be at the surgeon’s discretion. In both groups, the use of naso-gastric tubes, central lines, urinary catheter and post-operative contrast swallow is optional.

### Complications of surgery

Any major surgery involves the potential for complications; some are common to all abdominal operations, while others specific to bariatric surgery. Complications of abdominal surgery include infection, venous thromboembolism, haemorrhage, hernia and bowel obstruction (often many years after surgery).

A commonly reported complication of Band surgery is regurgitation. Other complications include ulceration; gastritis; erosion causing the band to slowly migrate through the stomach wall; slippage (rare); malposition of the band (rare with experienced practitioners); problems with the port and/or the tube connecting port and band; internal bleeding and infection.

Complications of Bypass surgery include anastomotic leak; anastomotic stricture; anastomotic ulcer and dumping syndrome. Bypass surgery can also lead to nutritional deficiencies. Patients are required to follow the surgeon’s instructions for food consumption, including the number of meals to be taken daily, adequate protein intake, and the use of vitamin and mineral supplements. Calcium supplements, iron supplements, protein supplements, multi-vitamins and vitamin B12 supplements are all very important to the post-operative Bypass patient.

### Study centres and surgeons

All centres will be surgical units carrying out at least 50 bariatric surgery operations per year and will have carried out a minimum of 250 Bypass and 250 Band procedures before entering patients into the trial. Participating surgeons will work within a specialist multi-disciplinary bariatric team with at least two surgeons. Phase 1 will take place in two UK centres. In Phase 2 recruitment will be extended into up to six more centres. Participating surgeons will have performed more than 100 laparoscopic Bypass procedures and more than 50 laparoscopic Band procedures for complex obesity.

### Quality control of surgery and follow-up appointments

Procedures will be video or digitally recorded and up to 10% sample of anonymised operations will be reviewed to ascertain adherence to mandatory and prohibited aspects of the surgery protocols. During Phase 1, a manual will be developed describing all the core mandated components of the surgical procedures, concomitant interventions and context and describe acceptable deviations (that is components that can be flexibly delivered) as well as those that are considered prohibited (unacceptable), as outlined above. This manual will then be used in Phase 2 to monitor quality control, fidelity to procedural steps, errors and near miss events. It is anticipated that the concomitant interventions that will be ‘manualised’ may include type of anaesthesia, and key elements of recovery pathways such as analgesia or intravenous fluid regimes.

Follow-up consultations will be according to specific protocols. This will be performed by a trained research nurse or surgeon. The patient will be interviewed by the research nurse to assess the amount of food they are able to eat, their appetite and whether they feel satisfied between meals. They will receive support and advice. For patients in the Band arm, if a fill is indicated, it will be carried out according to the local protocol and the patient will be tested for restriction. If there is too much restriction, fluid will be withdrawn. The band is filled progressively to reach the so-called ‘sweet spot’ of optimal restriction. Care is taken to try to avoid over-filling the band at any one time to avoid the problem of the need to withdraw fluid from the band urgently. Occasionally the port may not be accessible in the clinic and filling of the band may need to be done under X-Ray control. When this occurs it will be separately documented. However, fixing the port to the rectus sheath usually avoids the need to do this.

Participants in the Bypass arm will undergo similar supportive educational consultations performed by a trained research nurse or physician to help support and maintain a reduction in food intake and an increase in activity level. All patients will be interviewed about nutritional habits and behaviour and assessments of nutritional status performed in line with the American Association of Clinical Endocrinologists (AACE) guidelines [27]. Deficiencies identified will be treated and investigated in line with these guidelines. Persistent nausea, vomiting, dysphagia, ingestion, insufficient weight loss or weight gain will promote further investigation and additional reviews.

### Primary outcomes

There are two primary endpoints, i) the proportion of patients achieving a loss of greater than 50% of excess weight at three years, calculated as:

$$100\;\times \;\left[\mathrm{BMI}\;\mathrm{at}\;\mathrm{three}\;\mathrm{years}‒\mathrm{BMI}\;\mathrm{at}\;\text{randomisation}\right]/\left[\mathrm{BMI}\;\mathrm{at}\;\text{randomisation}‒25\right]$$

and ii) HRQOL at three years (EQ-5D health state score). BMI at baseline will be calculated using the participant weight recorded at baseline, after consent and before randomisation. The participants’ weight in kilograms (kg) will be measured on calibrated electronic clinic scales. Participants will be weighed fully clothed after removal of shoes, jackets and heavy items from pockets. Participants will stand with weight evenly balanced on both feet with their arms hanging loosely at their sides. Participants’ heaviest weight ever (both self-reported and the heaviest recorded in the participants’ medical records) will also be collected. Participants’ height at randomisation will be measured in centimetres with a calibrated stadiometer after removal of shoes.

The dual endpoint was chosen after careful consideration and discussion. It was chosen to be clinically meaningful to patients, surgeons and commissioners, that is combining effective weight loss with improved HRQOL. This is, however, a difficult area as there is no universally accepted definition of success (or failure) amongst patients having bariatric surgery and clinically meaningful improvements of quality of life and co-morbidity (for example, reductions in diabetes and sleep apnoea) can be achieved with as little as 20% total body weight loss [28]. In order to discriminate small differences between procedures in a way that will usefully inform the treatment choices, we wanted to set a criterion that would represent successful weight loss for the majority of patients. Therefore, we reviewed historic excess weight loss data for the two operations after three years for the lead site and chose 50% because this lay towards the ‘tail’ of the distribution, that is only 20% of patients experienced less than 50% excess weight loss.

### Secondary outcomes

These will include:

i) BMI over time adjusted for BMI at randomisation,

ii) percentage weight loss at three years:

$$\left[\left(\text{weight}\;\mathrm{at}\;\text{randomisation}‒\text{weight}\;\mathrm{at}\;\text{three}\;\text{years}\right)/\text{weight}\;\mathrm{at}\;\text{randomisation}\right]$$

iii) waist circumference at three years,

iv) time taken from randomisation to reach first loss of at least 50% of excess BMI,

v) time taken from first losing 50% excess BMI to first relapse (defined as weight re-gain such that the target of at least 50% of excess weight loss is no longer met),

vi) generic and symptom specific HRQOL to three years,

vii) resource use to three years,

viii) standard nutritional blood tests performed at each assessment including; full blood count, electrolytes, creatinine, glucose, HbA1c, liver function tests, iron, ferritin, vitamin B12, folate/red cell folate, lipid profile, 25-hydroxyvitamin D, calcium, parathyroid hormone,

ix) measures of 24-hour recall dietary intake using a standardised and validated interview process,

x) binge eating behaviour using a validated questionnaire,

xi) harms of surgery including peri-/post-operative complications, the need for re-operation and cross over between interventions,

xii) resolution of co-morbidities at three years, including sleep apnoea, non-alcoholic fatty liver disease, type-2 diabetes, hypertension and hyperlipidaemia, and

xiii) time to resolution of sleep apnoea, type-2 diabetes, hypertension and hyperlipidaemia.

### Assessment of secondary outcomes

The generic and disease specific measures include assessments with the SF12, EQ-5D [23], the Impact of Weight of Quality of Life-Lite (IWQOL-Lite) questionnaire [29,30], the Gastrointestinal Quality of Life Index (GIQLI, 36 items) [31], and the Hospital Anxiety and Depression Scale (HADS) [32]. Questionnaires administered at baseline before randomisation will be given to patients to complete themselves when they attend hospital. Questionnaires completed after surgery will be posted to participants by the coordinating centre to ensure that the follow-up time points are met. An option to log in to a secure web-site and complete the questionnaires on-line will also be provided. Reasons for the non-completion of questionnaires will be recorded. Missing or erroneous items on questionnaire measures will be handled according to the questionnaire developers’ scoring manuals. Reasons for withdrawal from the study, loss to follow-up or death (and cause of death) will be recorded. The dietary intake recall will be measured by trained research nurses, using repeat 24-hour recalls at baseline and single 24-hour recalls at all follow-up assessments. Sleep apnoea will be assessed using the STOP-BANG questionnaire at baseline and the Epworth Sleepiness Scale, completed at baseline and each research follow-up assessment [33]. Patients will be selected for sleep studies on the basis of history or a score of 5 or more using the STOP-BANG questionnaire [33]. An exploratory assessment of non-alcoholic fatty liver disease will be made at baseline and three years using the enhanced liver fibrosis test (ELF) [34].

### Assessment of co-morbidities

Resolution of co-morbidities will be assessed with the agreed definitions; resolution of obstructive sleep apnoea will be defined after repetition of sleep studies. The standard definition is less than five apnoea episodes per hour as assessed by polysomnography (sleep study). Remission of diabetes will be defined by criteria set out from a consensus meeting in Diabetes Care for remission after surgery [35] and HbA1c, fasting glucose and number of diabetes medications taken will be recorded at follow-up appointments. Remission is defined as achieving glycaemia below the diabetic range in the absence of active pharmacologic (anti-hyperglycaemic medications, immunosuppressive medications) or surgical (on-going procedures such as repeated replacements of endoluminal devices) therapy. A remission can be characterized as partial or complete. Partial remission is sub-diabetic hyperglycaemia (A1c not diagnostic of diabetes (<6.5%), fasting glucose 100 to 125 mg/dl (5.6 to 6.9 mmol/l)) of at least one year’s duration in the absence of active pharmacologic therapy or on-going procedures. Complete remission is a return to ‘normal’ measures of glucose metabolism (A1c) in the normal range, fasting glucose < 100 mg/dl (5.6 > mmol/l)) of at least one year’s duration in the absence of active pharmacologic therapy or on-going procedures. Remission from hypertension will be based on the international definition described in the metabolic syndrome, systolic blood pressure < 130 mmHg and diastolic < 85 mmHg without treatment. Standard remission of hyperlipidaemia will be defined as total cholesterol ≤ 5.0 mmol without cholesterol lowering treatments. We will also record the time to resolution of sleep apnoea, type-2 diabetes, hypertension and hyperlipidaemia. A 20 ml blood sample will also be taken at baseline and at three years for future investigations. The data collection schedule is summarised in Table 1.

**Table 1:** Data collection at the standard assessments

	**Pre randomisation**	**Post randomisation**						
		**Day of surgery**	**4 weeks**	**Months**				
				**3**	**6**	**12**	**24**	**36**
Weight	X	X	X	X	X	X	X	X
Height	X							
Blood pressure	X		X	X	X	X	X	X
Waist circumference	X		X	X	X	X	X	X
SF12	X		X		X	X	X	X
EQ-5D	X		X		X	X	X	X
IWQOL-Lite	X		X		X	X	X	X
GIQLI	X		X		X	X	X	X
HADS	X		X		X	X	X	X
Resource use questionnaires	X		X	X	X	X	X	X
Nutritional blood tests^c^								
Full blood count	X		X		X	X	X	X
Electrolytes	X		X		X	X	X	X
Creatinine	X		X		X	X	X	X
Fasting glucose	X		X		X	X	X	X
Lipids	X		X		X	X	X	X
HbA1c	X		X		X	X	X	X
Liver function tests	X		X		X	X	X	X
Iron, ferritin, vitamin B12	X		X		X	X	X	X
Folate/red cell folate	X		X		X	X	X	X
Lipid profile	X		X		X	X	X	X
25-hydroxyvitamin D	X		X		X	X	X	X
Calcium	X		X		X	X	X	X
Parathyroid hormone	X		X		X	X	X	X
Blood sample for future research	X							X
24-hour recall eating questionnaire	X			X	X	X	X	X
Binge eating questionnaire	X		X	X	X	X	X	X
Other co-morbidity								
Sleep apnoea								
STOP-BANG	X							
Epworth sleepiness scale	X			X	X	X	X	X
Non-alcoholic fatty liver disease^b^	X							X
National bariatric surgery registry			X		X	X	X	X
In-depth interviews^a^	X		X	X	X	X	X	X

Table showing data and blood samples collected at different stages of the participant’s pathway through the trial (X = data or sample collected).

^a^undertaken in a purposeful sample of participants.

^b^enhanced liver fibrosis test.

^c^for the assessment of co-morbidities.

### Assessment of resource use

The economic evaluation will follow established guidelines [36,37]. The main outcome measure will be quality adjusted life years (QALYs) using EQ-5D [23], to be administered at baseline, four weeks, six and 12 months and then at annual follow-up. Respondents will be assigned valuations derived from published UK population tariffs [38] and the mean number of QALYs per trial arm and incremental QALYs will be calculated. Data on percentage weight loss will act as an additional outcome measure. Data will be collected from the trial centres on health care resource use for surgery, follow-up appointments and treatments for any side effects. The costs for short term surgical complications such as peri-operative injury to adjacent organs and early post-operative morbidities such as staple leak or bleed will be estimated. The costs of longer term complications such as wound hernias, or the need for re-intervention or for cosmetic plastic surgery will be recorded.

Resource use will be measured in naturally occurring units; for example, staff time will be measured in terms of length of times for treatments and unit costs will be derived from nationally published sources where available and from trial centres. Collection of these details will allow micro-costing of the two surgical strategies. This is important information that we have identified as lacking, which can feed into NHS tariffs. Costs for contact with additional health care professionals as a result of surgery such as GP visits will be estimated.

### Participant follow-up

Follow-up will take place at four weeks, three, six, nine, 12, 24 and 36 months after surgery. Research data collection will not be required at the nine-month visit. Participants may have additional consultations outside these times as per the local protocol and participants’ requirements (expected to be up to ten appointments in the first two years). Active participation in the trial ends at 36 months. Thereafter, participants will be followed through the ‘Medical Research Information Service’ (NHS Information Centre) for mortality; the participant’s weight will be requested from the hospital on an annual basis.

### Sample size

The study size has been set at 614; allowing for a 15% loss to follow-up at three years, the target sample size is 724 patients. This will provide 90% power to test the two hypotheses that (a) Bypass will be non-inferior to Band in terms of the proportion of participants achieving an excess weight loss of at least 50% at three years and that (b) the HRQOL at three years for participants receiving a Bypass will be superior to the HRQOL for participants with a Band. The significance level for the non-inferiority hypothesis was set at 2.5% (one-sided) and for the superiority hypothesis it was set at 5% (two-sided).

The power calculation for the first hypothesis requires the estimation of two parameters, i) the total proportion of participants that are expected to have lost at least 50% of their excess weight at three years and ii) the difference in proportions achieving this target that would be considered clinically important (the non-inferiority margin). In By-Band it was assumed that 70% of patients will have lost ≥ 50% of their excess weight at three years and that a difference of ≥ 12% between the groups would be clinically important. The expected proportion of participants losing at least 50% of their excess weight at three years was estimated from the Taunton local database; for the sub-group with a BMI at surgery of between 40 and 60, 73% of Band and 75% of Bypass patients had lost at least 50% of their excess weight at three years. The non-inferiority margin was chosen on the basis of the opinions of the clinical team members and patient representatives.

The power calculation for the second HRQOL hypothesis, which for simplicity assumes the data will be analysed using analysis of covariance, requires the estimation of six parameters, the within group standard deviation, the difference in mean HRQOL that would be considered clinically important, the number of pre and post-surgery measures, and the correlations between pre and post-surgery scores and between repeated post-surgery scores. The estimates used were chosen on the basis of the published literature [39,40] and, in order to estimate correlations between different time points, on data from a surgical trial on spine injury. It was assumed that a small difference in mean HRQOL of 0.2 standard deviations would be clinically important. Conservative estimates of the correlation between repeated measures have been assumed (0.5 between pre and post measures, 0.75 between repeated post measures). The calculation is based on three post-surgery measures and assumes the treatment difference is similar at the three time points. However, the difference in HRQOL between groups may change over time. The calculation based on a single measure shows that the study will have 80% power to detect differences at individual time points. The sample size was calculated using Stata version 12.1 (Stata Corp LP, College Station, TX, USA).

### Statistical analyses

The analyses of the quantitative trial data will be based on intention-to-treat and will include all randomised patients. For the non-inferiority hypothesis a per-protocol analysis will also be carried out. Analyses will be adjusted for design factors included in the cohort minimisation. The proportion of patients with at least 50% excess weight loss at three years will be compared using mixed effects logistic regression. HRQOL scores (and other continuous outcomes measured at multiple time points) will be compared using a mixed linear regression model with baseline and post-surgery measures modelled jointly. Changes in treatment effect with time will be assessed by adding a treatment-by-time interaction to the model and comparing models with and without the interaction using a likelihood ratio test. Time-to-event outcomes will be compared using survival methods for interval censored data. Model fit will be assessed and alternative models and/or transformations (for example, to induce normality) will be explored where appropriate. Frequencies of adverse events will be described. Treatment differences will be reported with 95% confidence intervals. There is no intention to compare any outcomes between groups after Phase 1; the only analyses will be descriptive statistics to summarise recruitment to decide whether the trial satisfies the progression criteria.

The surgeon is not considered when randomly allocating participants to Bypass or Band as randomisation takes place 18 weeks before surgery. However, the allocation is stratified by centre and we will take the data structure into account, that is nesting of patients by surgeon and centre, in the primary analyses. One subgroup analysis is planned; outcomes will be described for patients with and without diabetes mellitus at baseline. Differences in treatment effect between the two subgroups will be tested by including an interaction term in the analysis model. This is a secondary analysis as the study is not powered to detect subgroup differences.

The health economic analysis will calculate the average cost and outcome on a per patient basis and, from this the incremental cost-effectiveness ratios for the different trial arms will be derived, producing an incremental cost per QALY and cost per percentage weight loss achieved. Probabilistic sensitivity analysis will be used to demonstrate the impact of the variation around the key parameters in the analysis on the baseline cost-effectiveness results. Results will be expressed in terms of a cost-effectiveness acceptability curve, which indicates the likelihood that the results fall below a given cost-effectiveness ceiling.

Decision modelling will be used to explore longer terms costs and effects for at least 20 years post-surgery. This will enable us to consider for instance longer term costs such as vitamin B12 replacement, calcium and vitamin D replacement for Bypass and follow-up for post-gastric surgery bone disease. Also cost savings as a result of a potential reduction or resolution in co-morbidities (for example, diabetes) will be explored.

### Changes to the protocol since first approved

Minor changes have been made. The exclusion of patients with a large hiatus hernia has been removed because participating surgeons are prepared to repair the hernia concurrently with the trial interventions. Post-discharge follow-up was changed from six weeks to four weeks on the recommendation of bariatric experts, to reflect the timing of the routine clinical follow-up post-surgery and a three-month follow-up was added for both groups. The Epworth sleepiness scale and the HADS questionnaire were added to improve assessment of secondary outcomes. Definition of resolution of sleep apnoea was revised to exclude discontinuation of the use of a continuous positive airways pressure (CPAP) machine and patient report, on the advice of the independent TSC. Recommended upper limits for the biliary and gastric limbs were changed from 100 cm and 200 cm respectively to 75 cm and 150 cm, respectively. Assessment of dietary intake at four weeks post-surgery was removed because patients are required to go on a six-week post-operative diet, so a four-week dietary recall is not useful.

### Development of a core outcome set to assess the benefits and adverse outcomes of bariatric surgery (BARIACT)

During Phase 1 of By-Band, a core outcome set to define the benefits and adverse surgical events will be developed using similar methodology as used by the OMERACT group [41]. The purpose of this is to ensure that future trials in obesity surgery report at the very least the core outcome set allowing for future evidence synthesis and cross study comparisons, as well as reducing reporting bias. Systematic literature reviews will identify all the current reported clinical outcomes of bariatric surgery (and their definitions). Outcomes reported by the National Bariatric Surgery Registry will be included. Qualitative interviews with surgeons and patients will identify additional potential outcomes of importance that are not identified from literature searches. Delphi methodology surveying surgeons and patients will reduce the potential list to a shorter list of outcomes to be discussed at the consensus meetings. In the Delphi survey, stakeholders will be asked to rate the importance of inclusion of each potential outcome in the core outcome set and two rounds will be undertaken to reduce the list according to pre-specified criteria. Each Delphi round will be analysed to identify key or redundant items from the list. A consensus meeting will be convened with key stakeholders at the same time as a TSC meeting to discuss the survey results and to perform further anonymised rating of the importance of retained items. The work will link with the COMET initiative and the MRC ConDuCT Hub for trials methodology research [42,43]. The final core set of outcomes of bariatric surgery is expected to be fewer than ten items.

## Discussion

The By-Band study will examine the effectiveness and cost-effectiveness of the two most commonly performed surgical procedures for complex obesity. It has integrated qualitative research to overcome the likely barriers to recruitment related to surgeon and patient preferences [44]. The By-Band study, however, is an open surgical trial in which participants, clinicians and other hospital staff caring for participants will not be ‘blind’ to their allocation. This is because of the need for adjustment of gastric bands with injection of saline into the subcutaneous port. It was considered too difficult to put in place an equivalent ‘sham’ procedure for participants having Bypass (given patients’ knowledge about the operations). Moreover, using a sham control might have removed one of the influences on HRQOL of Bypass, namely less intensive hospital follow-up. The study, therefore, is at risk of bias, particularly performance and detection bias [45]. The following measures have been put in place to minimise the potential for bias: i) concealed randomisation to protect against selection bias, ii) standard protocols (and monitoring of adherence) for follow-up after both procedures to minimise the risk of performance bias arising from carers differentially providing co-interventions, iii) blinding of the assessor undertaking measurements of all outcomes at the primary endpoint (three years) to minimise detection bias; iv) other outcomes defined as far as possible on the basis of objective criteria (for example, biochemical markers will be measured by an independent laboratory technician at the local hospital, without knowledge of treatment allocation). Every effort to keep in touch with participants (through annual assessment; checking on change of address, and so on) will be made especially if a participant misses an annual follow-up assessment to minimise attrition bias. The success of blinding will be tested and reasons for unblinding recorded. Self-completion HRQOL measures will inevitably be susceptible to bias although expectations about any effects of the different procedures prior to surgery are likely to be reduced through optimising information given prior to surgery (informed by the qualitative research) and it is expected that these will wane with follow-up, so participants will not have strong differential expectations of the treatments after three years.

The joint hypothesis of non-inferior weight loss and superior HRQOL with Bypass was chosen to reflect expert opinion on the benefits and risks of the two procedures. It is recognised that the weight loss profile is different for the two procedures; there is rapid weight loss with Bypass, which may be difficult to maintain in the longer term, whereas with Band the weight loss is more gradual; observational data suggests at three years the excess weight loss is comparable, hence the non-inferiority hypothesis. The likelihood of longer term complications (such as the need for revision surgery), however are greater for Band than Bypass and this is why it is hypothesised at three years there will be superior HRQOL with Bypass.

The trial will be analysed on an intention-to-treat basis, that is outcomes will be analysed according to the treatment allocation, irrespective of future management and events, and every effort will be made to include all randomised participants. We recognise that a per-protocol analysis is advocated for non-inferiority hypotheses and we will also conduct an analysis by surgery received of the proportion achieving at least 50% excess weight loss at three years. However, we anticipate that cross-overs will be few. A detailed statistical analysis plan will be prepared in advance of any formal analysis of the study data, which will be discussed with members of both the TSC and the Data Monitoring and Safety Committee. Follow-up for the outcome measures during the participant’s stay in hospital should be complete for all participants.

In Phase 1, we will review practice and incidence of sleeve gastrectomy as it is possible that this may have increased since the start of the study. If so, consideration will be given to incorporating sleeve gastrectomy into the trial design by discussion with the funding body and the TSC.

## Trial status

The trial opened to recruitment in one centre in November 2012 and in the other centre in February 2013. Interviews with the study teams have taken place, consultations have been audio-recorded, analysed and the results fed back to the study team. Trial participation has increased following this feedback. Recruitment in the two Phase 1 centres is on-going.

## Abbreviations

AACE: American Association of Clinical Endocrinologists; BMI: body mass index; CI: chief investigator; CPAP: continuous positive airways pressure; CONSORT: Consolidated Standards of Reporting Trials; ELF: liver fibrosis test; EQ-5D: health related quality of life; GIQLI: Gastrointestinal Quality of Life Index; HADS: Hospital Anxiety and Depression Scale; HRQOL: Health Related Quality of Life; HTA: Health Technology Assessment; IWQOL-Lite: Impact of Weight of Quality of Life-Lite; NHS: National Health Service; NICE: National Institute for Health and Care Excellence; NIHR: National Institute for Health Research; PI: principal investigator; QALY: Quality Adjusted Life Years; RCT: randomised controlled trial; TMG: Trial Management Group; TSC: Trial Steering Committee; UK: United Kingdom.

## Competing interests

The authors declare that they have no competing interests.

## Authors’ contributions

CAR: study design, preparation and drafting of study protocol, sample size and statistical analysis plan, drafting of manuscript. RW: study design, preparation of study protocol, definition of surgical interventions, review of manuscript. Lead surgeon in trial. JB: study design, preparation of study protocol, definition of surgical interventions, review of manuscript. Principal investigator in Southampton. JD: design of integrated qualitative study, preparation of study protocol, review of manuscript. BCR: study design, preparation and drafting of study protocol, strategies to minimise bias, review of manuscript. SW: study design, preparation and drafting of study protocol, design of health economic component, review of manuscript. RA: study design, preparation and drafting of study protocol, definition of clinical endpoints, review of manuscript. Principal investigator in Taunton. JLT: study design, preparation and drafting of study protocol, design of dietary assessments, review of manuscript. PR: study design, preparation of study protocol, review of manuscript. DM: preparation of study protocol, review of manuscript. HN: preparation of study protocol, review of manuscript. JK: preparation of study protocol, review of manuscript. GM: preparation of study protocol, review of manuscript. KP: preparation of study protocol, review of manuscript. SP: preparation of study protocol, review of manuscript. NB: preparation of study protocol, review of manuscript. MP: preparation of study protocol, review of manuscript. TP: preparation of study protocol, review of manuscript. JMB: study concept, study design, preparation of study protocol, drafting and review of manuscript. Chief investigator of the trial. All authors read and approved the final manuscript.

## Authors’ information

Independent Trial Steering Committee Members:

Professor Joanna Coast (chair), School of Health and Population Sciences, University of Birmingham.

Professor John Dixon, Bariatric Physician, Baker IDI Heart and Diabetes Institute, Melbourne, Australia.

Dr Charlotte McCaie, Patient Representative.

Miss Sally Norton, Consultant Bariatric Surgeon, North Bristol NHS Trust.

Professor Dr Michel Suter, Hôpital du Chablais, 1860 Aigle Switzerland.

Independent Data monitoring and safety committee members:

Professor Craig Ramsay (chair), Health Services Research Unit, University of Aberdeen.

Professor Nick Finer, Consultant in Bariatric Medicine, University College Hospital, London.

Dr Torsten Olbers, Senior Physician, Sahlgrenska University Hospital, Gothenburg, Sweden.
